# X Chromosome: Expression and Escape

**DOI:** 10.1371/journal.pgen.1000724

**Published:** 2009-11-20

**Authors:** John Parsch

**Affiliations:** Department of Biology, University of Munich, Planegg-Martinsried, Germany; Fred Hutchinson Cancer Research Center, United States of America

Although there are some obvious differences between the X chromosome and the autosomes—such as the X chromosome being present in only one copy in males—the two types of chromosome are remarkably similar in their cytological appearance and gene density [1]. Genomic and transcriptomic studies of *Drosophila*, however, have revealed two major differences in gene content between the X chromosome and the autosomes. First, there is an excess of functional, duplicate genes that have moved from the X chromosome to the autosomes (Figure 1A) [2]. Second, there is a paucity of genes with male-biased expression on the X chromosome (Figure 1B) [3]. A hypothesis that could explain these observations was proposed by Betrán and colleagues in 2002 [2] and is based on the phenomenon of meiotic sex chromosome inactivation (MSCI, see below). Since then, there has been controversy about whether or not MSCI occurs in *Drosophila* and, if it does, what role it plays in shaping the gene content of the X chromosome. In this issue of *PLoS Genetics*, Vibranovski et al. [4] present a detailed analysis of gene expression across three stages of *Drosophila* spermatogenesis. Their results support the occurrence of MSCI and suggest that its effect on germline expression of X-linked genes promotes the selective maintenance of autosomal duplicates arising from X-linked genes.

**Figure 1:**
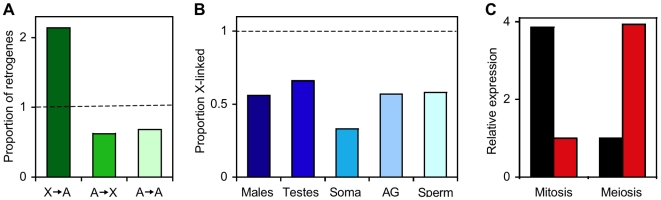
The X chromosome and autosomes of *Drosophila melanogaster* differ in retrogene and male-biased gene content. (A) Relative proportions of retrogenes that have moved from the X chromosome to the autosomes (X→A), the autosomes to the X chromosome (A→X), or from one autosome to another (A→A). The dashed line indicates the expected (random) proportion. Data are from [2]. (B) The proportion of X-linked genes (relative to the random expectation; dashed line) for male-biased genes of five categories, including those expressed in whole adult males, testes, somatic male tissues, accessory glands (AG), and sperm. Data are from [3],[11],[18]. (C) Complementary expression of X-linked parental genes (black) and their autosomal retroposed copies (red) during the mitosis and meiosis stages of spermatogenesis. Data are from [4].

## Meiotic Sex Chromosome Inactivation (MSCI)

In an insightful paper from 1972, Lifschytz and Lindsley [5] proposed that, in species with XY sex determination, the X chromosome is functionally inactivated during spermatogenesis. This process is now commonly referred to as MSCI. A consequence of MSCI is that X-linked genes cannot be expressed for as long as autosomal genes during the course of spermatogenesis, as transcription ceases when the X chromosome is inactivated. Thus, on average, one would expect autosomal genes to show higher levels of expression in the male germline than X-linked genes. This especially should be the case for genes expressed in the later stages of spermatogenesis, after X-inactivation has taken place. Such an imbalance in expression might favor duplicate gene copies that “escape” the X chromosome and move to the autosomes. The new autosomal copies would be able to be expressed at higher levels during the later stages of spermatogenesis, and, if this expression is beneficial to the organism, the new copies would be retained by selection more often than other types of gene duplicates (for example, those moving from autosome to autosome).

Although MSCI has been demonstrated in several taxa [6], its occurrence in *Drosophila* has been controversial, and recent studies have produced conflicting results. Microarray analyses of whole male flies and whole testes found no evidence for a universal reduction in the expression of X-linked genes, which would have been expected if MSCI had a global effect on X-chromosome gene expression [7]. In contrast, an experimental test of MSCI, using a testis-expressed transgenic reporter gene mobilized to various X-linked and autosomal locations, provided strong evidence for inactivation of the X chromosome during spermatogenesis [8]. To avoid some of the shortcomings of previous studies and directly test for MSCI on a genome-wide scale, Vibranovski et al. performed a global analysis of gene expression in testes that were carefully dissected into three different stages of spermatogenesis.

## Stage-Specific Expression Profiling

In *Drosophila*, spermatogenesis proceeds from the apical to the distal part of the testis. The apical region is comprised mainly of mitotic cells, the proximal region is enriched for meiotic cells, and the distal region contains mainly post-meiotic cells. To investigate gene expression at different stages of spermatogenesis, Vibranovski et al. painstakingly dissected hundreds of testes into these three regions, which they labeled “mitosis”, “meiosis”, and “post-meiosis”. Inactivation of the X chromosome is expected to occur between the first two of these stages.

A microarray analysis of RNA extracted from cells of the three different stages provides the first global analysis of gene expression across *Drosophila* spermatogenesis. This, in itself, represents a major accomplishment that will be a valuable resource for many researchers. Reassuringly, genes that previously were known to be expressed at the different stages show the expected pattern in the microarray data. Using a novel Bayesian analysis method, Vibranovski et al. show that a significantly greater proportion of autosomal genes than X-linked genes have enriched expression at the meiosis stage. Furthermore, they show that there is an under-representation of X-linked testis-expressed genes at the meiosis stage but not at the mitosis stage. Both of these observations are consistent with the occurrence of MSCI.

## The Genomic Distribution of Retrogenes and Male-Biased Genes

Does MSCI contribute to the excess of functional, duplicate genes that have moved from the X chromosome to the autosomes? To investigate this, Vibranovski et al. looked at the expression profiles of retrotransposed genes (“retrogenes”—those that duplicated through a reverse-transcribed mRNA intermediate) and compared them to those of their original “parental” genes. In most cases, they found that the new autosomal copies had higher expression at the meiosis stage than at the mitosis stage, while their X-linked parental genes showed the opposite pattern (Figure 1C). Such complementary gene expression would be expected if selection favored genes that moved from the X chromosome to the autosomes in order to escape MSCI and increase their expression during the later stages of spermatogenesis. It is likely that this effect of MSCI is relevant not only to *Drosophila*, but also to other taxa with XY sex determination, as a similar excess of retrogene movement from the X chromosome to the autosomes has been observed in mammals [9],[10].

Can MSCI explain the genomic distribution of genes with male-biased expression? Here, the answer is not so clear. The strongest argument against MSCI as a major factor shaping the genomic distribution of male-biased genes in *Drosophila* is that genes with male-biased expression in somatic tissues also are under-represented on the X chromosome (Figure 1B) [3],[7],[11]. An alternative hypothesis involving the differential accumulation of sexually antagonistic mutations (those that are beneficial to one sex but detrimental to the other) on the X chromosome and autosomes may better explain the genomic distribution of male-biased genes [12]–[14]. However, critical parameters regarding the abundance, dominance, and sex-specific effects of sexually antagonistic mutations, as well as their relationship to sex-biased gene expression, remain unknown. Furthermore, these parameters are likely to differ among taxa, as the mammalian X chromosome shows an enrichment of genes expressed in early spermatogenesis and in somatic reproductive tissues [15]–[17]. Future studies are needed to determine the relative contributions of MSCI and sexual antagonism (or other factors) to differences in gene content between the X chromosome and the autosomes.
